# The Efficacy of Cone-Beam CT-Based Perfusion Mapping in Evaluation of Tissue Perfusion in Peripheral Arterial Disease

**DOI:** 10.3390/jcm10050947

**Published:** 2021-03-01

**Authors:** Ran Kim, Sun Young Choi, Yeo Ju Kim

**Affiliations:** 1 Department of Radiology, Ewha Womans University Mokdong Hospital, College of Medicine, Ewha Womans University, 1071, Anyangcheon-ro, Yangcheon-gu, Seoul 07985, Korea; rankim1001@gmail.com; 2 Department of Radiology, Hanyang University Hospital, College of Medicine, Hanyang University, 222-1, Wangsimni-ro, Seongdong-gu, Seoul 04763, Korea; kimyeojurad@gmail.com

**Keywords:** peripheral arterial disease, critical limb ischemia, percutaneous transluminal angioplasty, parenchymal blood volume, cone-beam CT-based perfusion mapping

## Abstract

This study investigated the use of cone-beam computed tomography (CBCT)-based perfusion mapping during percutaneous transluminal angioplasty (PTA) to predict clinical outcome in the peripheral arterial disease (PAD). From January 2016 to March 2020, 43 patients (28 male, 15 female; mean age, 69) with 51 limbs, who underwent PTA with CBCT-based foot perfusion mapping for PAD were included. Parenchymal blood volume (PBV) of foot was measured. Clinical response was investigated based on medical records. Predictive value for clinical success was evaluated using multiple logistic regression with C-statistics. Two reviewers visually assessed the improvement on angiography and CBCT-based foot perfusion mapping; inter-observer agreement of clinical success between the two were measured. Technical and clinical success rate of PTA was 90.8% and 68.6%, respectively. In multiple logistic regression, the maximum value of PBV (PBV_max_) on perfusion mapping after PTA was significant (*p* = 0.03) for evaluating clinical success with the highest C-statistic (0.84). Using a cutoff of 235.7 mL/L for PBV_max_ after PTA, area under curve for prediction of clinical success was 0.664, and sensitivity and specificity were 71.4% and 68.8%, respectively. Consistency in prediction of clinical success between the two reviewers was almost perfect for CBCT-based foot perfusion mapping.

## 1. Introduction

A population of more than 200 million suffers from peripheral arterial disease (PAD); it is a significant disease spectrum associated with high morbidity and mortality [1]. Patients experience varying symptoms ranging from claudication to critical limb ischemia (CLI) such as ischemic rest pain, ulcer, or gangrene [2,3,4,5]. In patients who have PAD coexisting with diabetes, the severity of PAD is greater, and the risk of major amputation and mortality rate are higher than in those without diabetes [6,7].

In the past, the treatment for PAD was either medication or surgical amputation. However, with the recent development in percutaneous transluminal angioplasty (PTA) and its advantage of minimal invasiveness and high success rate, PTA has become a representative treatment in PAD [8,9,10,11]. However, despite the technical success of PTA, cases with clinically equivalent results are often not obtained. The clinical outcomes in individuals may vary even with the same treatment, due to the presence or absence of an underlying medical condition, such as diabetic mellitus or various other systemic conditions [12]. Moreover, fundamental doubts regarding the accurate evaluation of the results of the PTA procedure through angiography exist. The basis for performing PTA in PAD patients is the restoration of the inflow of stenosis or occlusion of the arterial system, thereby increasing vascular flow to the tissue, and ultimately alleviating symptoms and healing wounds. Therefore, in order to predict clinical success through analysis of technical results, it is necessary to ultimately evaluate changes in tissue perfusion through the procedure. Two-dimensional angiography can only evaluate visually observable vascularity [13]. Therefore, if there is a method that can accurately measure the periprocedural tissue perfusion change during the PTA procedure, it may be correlated with the clinical outcome of revascularization and can be helpful to predict the clinical course and determine the treatment direction.

Traditionally, efforts have been made to measure tissue perfusion through methods such as conventional computed tomography (CT), magnetic resonance imaging, ultrasonography, and scintigraphic methods; since these techniques are employed after endovascular treatment, a change in treatment plan during the procedure is difficult. Moreover, some of them have limitations in terms of accuracy, reliability, and increased medical cost [14,15,16]. If accurate perfusion imaging can be obtained during the endovascular treatment, a prompt and more accurate treatment plan can be formulated. Recent advancements in technology have facilitated perfusion mapping by angiography equipment guided by a cone-beam CT (CBCT) [17,18,19,20]. Here, we evaluate the effectiveness of CBCT-based foot perfusion mapping obtained during the PTA procedure, to predict treatment response and clinical outcome.

### 1.1. Patients

Our institutional review board approved this retrospective study (No. 2020-06-003) and waived the requirement for informed consent. From January 2016 to March 2020, consecutive PAD patients who underwent PTA using a CBCT system equipped with post-processing software (Artis Q; Siemens Healthcare, Erlangen, Germany), were evaluated. If PTA procedure, during the study period, was repeated in a patient in the same limb, only data from the first examination were included. During the period, 54 patients with 62 limbs underwent PTA, with CBCT perfusion mapping during the procedure. Among them, 11 patients were excluded; four were treated with PTA for thromboembolism, three were lost to follow-up, and raw data of CBCT was lost for four. Thus, 43 patients with 51 limbs were included in this study. Clinical follow up was also reviewed retrospectively from the hospital electronic medical chart. Patient characteristics are summarized in Table 1.

The PTA was indicated in our hospital for clinically and radiologically suspicious PAD, and for certain other cases, if deemed clinically necessary. The clinical diagnosis criteria for PAD were: (1) abnormal ankle-brachial systolic pressure index (ABI) and (2) signs and symptoms indicative of extremity ischemia such as claudication or rest pain, skin discoloration, and an ulcer on extremity. The radiologic diagnostic criteria for PAD were: (1) More than 50% of stenosis above the knee region on ultrasonography or CT angiography; (2) more than two poorly delineated arteries in a below the knee (BTK) lesion on ultrasonography or CT angiography; and (3) if there was a wound, any related, poorly delineated blood vessel in BTK lesion on ultrasonography or CT angiography.

### 1.2. Endovascular Treatment and CBCT Acquisition

**Figure 1:**
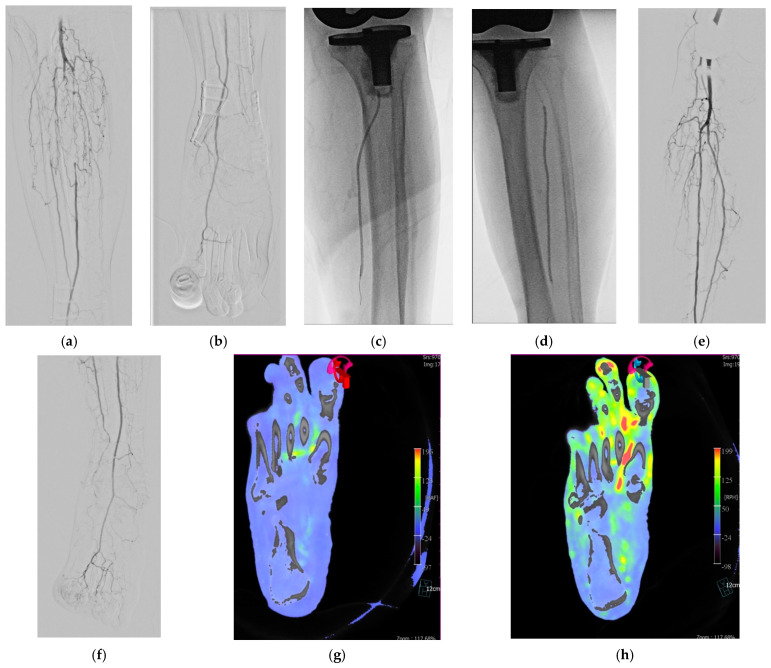
A 73-year-old woman with peripheral arterial disease underwent percutaneous transluminal angioplasty for the first and second toe wound. (**a**,**b**) Initial angiography shows long segmental occlusion of left anterior tibial artery (ATA) and posterior tibial artery (PTA) and total occlusion of left peroneal artery with decreased flow to the foot including first and second toe. (**c**,**d**) Recanalization with 0.014-inch guidewire and balloon angioplasty was done with a 2 mm–15 cm balloon catheter. (**e**,**f**) Completion angiography shows patent left ATA and PTA and increased flow to the foot especially first and second toe. (**g**,**h**) Cone-beam computed tomography-based foot perfusion mapping before (**g**) and after (**h**) angioplasty clearly shows markedly increased perfusion of the foot, especially first and second toe.

**Figure 2:**
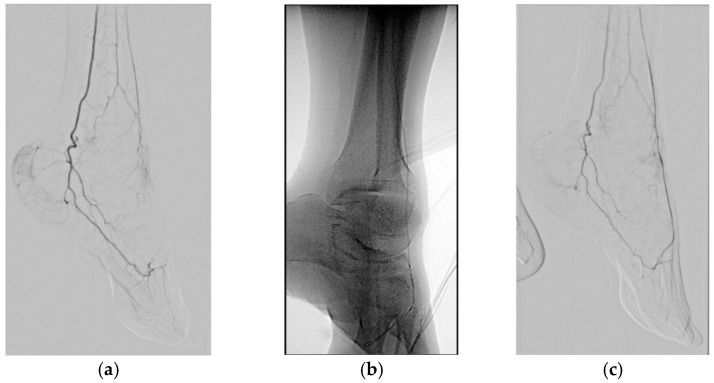

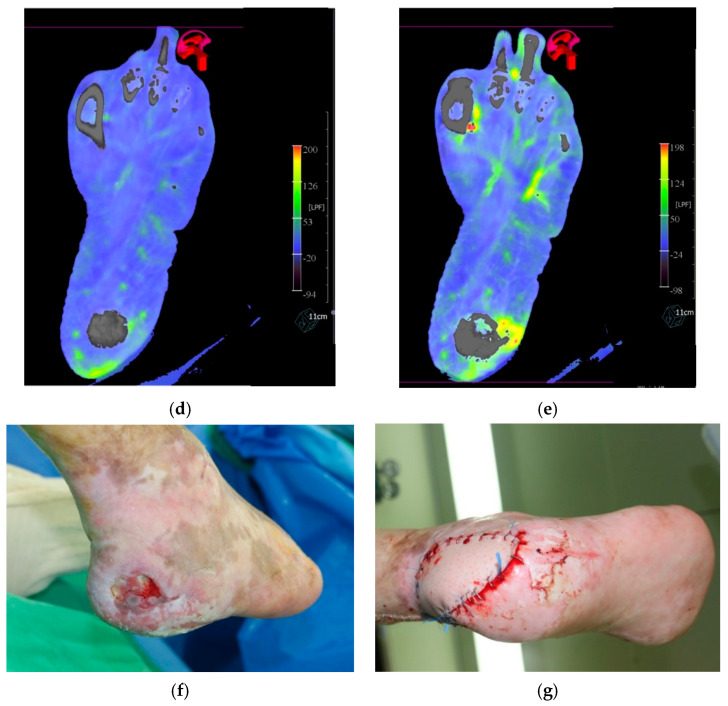
A 58-year-old man with peripheral arterial disease underwent percutaneous transluminal angioplasty (PTA) for heel wound. (**a**) Initial angiography shows total occlusion of left dorsalis pedis artery. (**b**) Recanalization with 0.014-inch guidewire and balloon angioplasty was done with a 1.5 mm–4 cm balloon catheter. (**c**) Completion angiography shows patent left dorsalis pedis artery and no significant change of flow to heel side. (**d**,**e**) Cone-beam computed tomography-based foot perfusion mapping before (**d**) and after (**e**) PTA clearly shows markedly increased perfusion of the foot, especially heel side. (**f**) Photography shows a wound on the heel side. (**g**) Patient underwent reconstruction surgery with an anterolateral thigh flap two weeks later after PTA without undergoing amputation.

The PTA procedure for all patients was performed, in an angiographic suite equipped with a flat panel detector C-arm angiographic system (Artis Q), by an interventional radiologist with 14 years of experience. Using ultrasound, depending on the location of lesions, percutaneous vascular access was achieved by an antegrade or retrograde puncture of the common femoral artery (CFA). The main principle of PTA was followed: (1) an intraluminal approach rather than subintimal approach through all target arterial segments. (2) Iliac artery; plain old balloon angioplasty (POBA) rather than primary stenting. (3) CFA; atherectomy with POBA. (4) Superficial femoral artery (SFA); drug-coated balloon (DCB) angioplasty followed by stent placement in case of dissection or suboptimal result, rather than primary stent placement or drug-eluting stent placement. (5) Popliteal artery (PA); DCB or POBA. (6) BTK arteries; POBA (Figure 1a–d and Figure 2a,b).

CBCT-based foot perfusion mapping was routinely obtained before and after the angioplasty in PAD patients. However, if the patient failed to cooperate during the procedure due to irritability, or the patient’s position was head to toe due to combined iliac lesions, CBCT-based foot perfusion mapping was not obtained.

The CBCT parameters utilized were 0.5° increment, 211° circular trajectory, 512 × 512 matrix in projections, and 48-cm field-of-view in two-dimensional (2D) raw data. To obtain the CBCT scan, the acquisition protocol consisted of two rotations: an initial unenhanced rotation (mask run) followed by an injection of contrast medium, and then a second contrast-enhanced rotation (fill run) after an appropriate scan delay [19,20,21]. Each run took about 5 s, and an interval of about 1 s was required between each step. Therefore, a mask run without contrast medium took 5 s. During the return run, a scan delay was implemented, and an additional delay time was used if necessary. The scan delay to obtain a fill run in CBCT acquisition was manually adjusted according to the previously performed angiography within a range of 6–10 s. Overall, 17–20 s were required to obtain the images. For contrast, a 4-F catheter tip was positioned at the normal segment of peripheral artery and 33% diluted iodine contrast medium in normal saline at a rate of 7 mL/s, 6 mL/s, 5 mL/s, and 3 mL/s was injected for common and external iliac artery, CFA, SFA, and PA, in that order, for a duration of scan delay time and fill run. Acquired data was sent to the workstation (syngo DynaPBV Body; Siemens Healthcare). CBCT-based foot perfusion mapping was obtained automatically from the CBCT scan using the post-processing software. Foot perfusion mapping was reconstructed by axial images of the long axis of the foot, based on a line connecting the lowermost part of the calcaneus bone and the head of the third metatarsal bone in parallel (Figure 1g,h and Figure 2d,e).

When the obvious vasospasm was noted at the completion of angiography, an intra-arterial vasodilator, in other words, 100 μg of nitroglycerin (glyceryl trinitrate, Pohl-Boskamp, Hohenlockstedt, Germany) was injected and, CBCT-based foot perfusion mapping was done after the completion of angiography.

Patients were given 100 mg aspirin and 75 mg clopidogrel for 3 days before the procedure. A bolus dose of 3000 U of heparin was administered immediately before starting the procedure; and if the procedure lasted for > 1 h, an additional dose of 1000 U of heparin was given every hour. Upon discharge, patients were advised 100 mg aspirin for life and 75 mg clopidogrel daily for 1–3 months. When a drug-eluting stent was placed, patients were recommended to continue the treatment for 3–12 months.

### 1.3. Definitions

The technical success of angiography after angioplasty was defined as <30% of residual stenosis above the popliteal lesion, and recanalization of target vessel with balloon angioplasty in BTK arteries. The clinical success, on reviewing hospital electronic medical charts, was defined as total or partial disappearance of symptoms such as claudication, rest pain, and “others”, with an increase in ABI; and healing of wound, favorable outcome after flap surgery without amputation, or planned minor amputation with an increase in ABI in wound patients (Figure 2f,g).

### 1.4. Image Analysis

Two experienced radiologists, who were blinded to the PTA procedure, on comparing the data acquired before and after angioplasty, reviewed and visually scored the improvement noted in arterial flow on angiography and tissue perfusion on CBCT-based foot perfusion mapping independently, after achieving only basic consensus. The following 4-point scale was used for image analysis: 0, worse; 1, no change; 2, improved <50%; 3, markedly improved ≥50%. A score of 0 or 1 was considered a negative decision on improved flow or perfusion, whereas a score of 2 or 3 was considered positive. Correlations between clinical outcome evaluation with visual scoring by imaging modalities after percutaneous transluminal angioplasty and clinical outcome are summarized in Table 2.

The improved blood flow at the target lesion after angioplasty was defined as an increase in contrast enhancement on angiography and increased perfusion on CBCT-based foot perfusion mapping. Mean value of parenchymal blood volume (PBV_mean_) and maximum value of parenchymal blood volume (PBV_max_) was obtained as perfusion parameters at the level of long axis containing the maximum area of the target lesion in wound patients, and the long axis of the sole in other patients. Analysis of perfusion parameters before and after angioplasty was performed by the interventional radiologist, who performed the PTA procedure.

### 1.5. Statistical Analysis

Diagnostic performance of clinical success on angiography, and CBCT-based foot perfusion mapping before and after angioplasty was evaluated for each reviewer. PBV_mean_ and PBV_max_ of foot before and after angioplasty were compared using Wilcoxon signed rank sum test. Receiver operating characteristic (ROC) curve analysis was performed to determine a cut-off value of perfusion factor for the prediction of clinical success. Multiple logistic regression analysis, which was adjusted by amputation factor, was performed using C-statistics and odds ratio with 95% confidence interval to evaluate the ability to determine clinical success based on the imaging type. Inter-observer agreement of clinical success with binary outcome (negative decision for improved flow or perfusion (score of 0 or 1) vs. positive decision (score of 2 or 3)) on angiography and CBCT-based foot perfusion mapping, between the two reviewers, was measured with use of the κ coefficient. The strength of agreement based on κ values was interpreted as follows: slight, 0.00–0.20; fair, 0.21–0.40; moderate, 0.41–0.60; substantial, 0.61–0.80; and almost perfect, 0.80–1.00 [22]. A *p* value of <0.05 was considered to be statistically significant. Statistical analysis was performed using SAS version 9.4 software (SAS Institute Inc., Cary, NC, USA).

## 2. Results

### 2.1. Treatment Outcome after Endovascualr Treatment

A total of 185 lesions in 51 limbs were treated with PTA, with the technical success rate of 90.8% (168/185). The technical success rate for each lesion was as follows: Common iliac artery, 100% (1/1); external iliac artery, 100% (1/1); CFA, 100% (1/1); SFA, 100% (29/29); PA, 97% (28/29); tibio-peroneal trunk, 100% (13/13); anterior tibial artery, 88.2% (30/34); posterior tibial artery, 81.3% (26/32); peroneal artery, 92% (23/25); and dorsalis pedis artery and medial or lateral plantar arteries, 80% (16/20) (Figure 1e,f and Figure 2c). Detailed individual angioplasty procedures according to the treatment segments are summarized in Table 3. The clinical success rate was 68.6% (35/51). The clinical success rate for each indication was as follows: rest pain, 100% (1/1); claudication, 100% (17/17); wound, 65.4% (17/26); and others, 0% (0/7). The reason for the procedure in “others” was as follows: an incidental finding on lower extremity CT angiography, that was done on physician’s request (5/7), tingling sensation, later discovered to be caused by other reason (1/7), and for quick recovery of the multiple lower extremity fractures, with an incidentally found vascular stenosis on lower extremity CT angiography taken after surgery (1/7). Six patients underwent reintervention in the same limb; PTA segments were as follows: SFA (*n* = 1), anterior tibial artery (*n* = 4), posterior tibial artery (*n* = 1), peroneal artery (*n* = 1), and dorsalis pedis artery (*n* = 2). Eleven patients underwent amputation after PTA; toe amputation (*n* = 7), midtarsal amputation (*n* = 1), and BTK amputation (*n* = 3). Regardless of PTA results, two patients died during the follow-up period: acute cerebral infarction in one patient and septic shock caused by aspiration pneumonia in the other. During the endovascular treatment, 100 mcg nitroglycerin was injected into three patients.

### 2.2. Diagnostic Performance of Clinical Outcome Evaluation

Accuracy, sensitivity, positive predictive value (PPV), and negative predictive value (NPV) of visual scoring of CBCT-based foot perfusion mapping was higher than those of angiography for both reviewer 1 and reviewer 2. Only specificity of visual scoring of angiography was relatively higher than that of CBCT-based foot perfusion mapping. Overall diagnostic performance by imaging types to evaluate the clinical success are summarized in Table 4.

### 2.3. Quantitative Analysis of Perfusion Factors before and after Angioplasty

**Figure 3:**
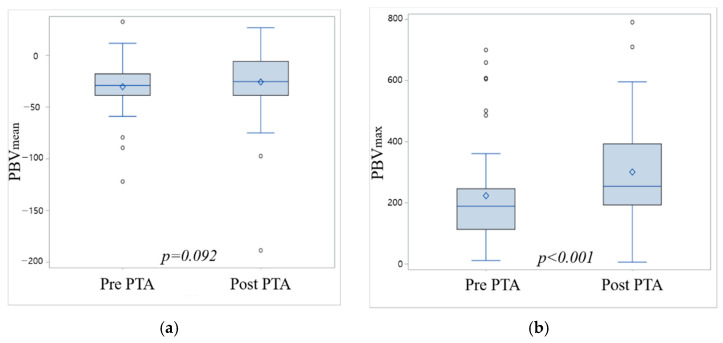
Quantitative analysis of perfusion parameters, measured on axial images of cone-beam CT-based perfusion mapping, showing the long axis of the foot demonstrating parenchymal blood volume (PBV) before and after percutaneous transluminal angioplasty (PTA). (**a**) PBV_mean_ of foot was not significantly increased after angioplasty (median value −29.11 before PTA to −25.44 after PTA, *p* = 0.092). (**b**) PBV_max_ of foot was significantly increased after angioplasty (median value 189 before PTA to 254 after PTA, *p* < 0.001).

**Figure 4:**
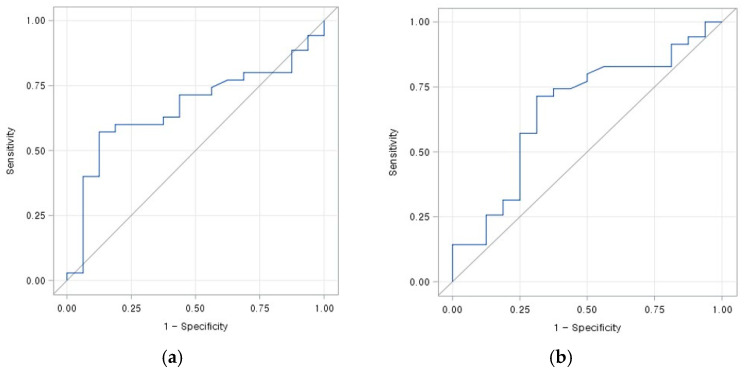
Receiver operating characteristic curve analysis of perfusion factors of foot to predict clinical success on cone-beam CT–based foot perfusion mapping obtained before and after percutaneous transluminal angioplasty for peripheral arterial disease. (**a**) Pre-PBV_max_ of foot (area under curve = 0.663, cutoff value = 215, sensitivity = 57.1%, specificity = 87.5%). (**b**) Post-PBV_max_ of foot (area under curve = 0.664, cutoff value = 235.7, sensitivity = 71.4%, specificity = 68.8%).

PBV_max_ of foot was significantly increased and PBV_mean_ of foot was not increased to significance after angioplasty (Figure 3). ROC curve analyses for PBV_max_ of foot before and after angioplasty are shown in Figure 4. Using a cutoff of 215 mL/L for PBV_max_ of foot before angioplasty, area under curve (AUC) for prediction of clinical success was 0.663, and sensitivity and specificity was 57.1% and 87.5%, respectively. Using a cutoff of 235.7 mL/L for PBV_max_ of foot after angioplasty, AUC for prediction of clinical success was 0.664, and sensitivity and specificity was 71.4% and 68.8%, respectively.

### 2.4. Ability to Predict Clinical Outcome According to Imaging Type and Perfusion Parameter

Multiple logistic regression analysis, with adjustment for amputation, to assess the ability of imaging modalities to predict clinical success is shown in Table 5. PBV_max_ of perfusion mapping after angioplasty, for evaluating clinical success with the highest C-statistic (0.84), was statistically significant. PBV_max_ of perfusion mapping before angioplasty and visual assessment of CBCT-based foot perfusion mapping, for evaluating clinical success with high C-statistic (0.82 and 0.81 in order), were also excellent. Angiography and PBV_mean_ of perfusion mapping before and after angioplasty for prediction of clinical success were not statistically significant.

### 2.5. Consistency of Visual Assessment

Consistency of visual assessment by means of the 4-point scale by using CBCT-based foot perfusion mapping had a higher degree of agreement, than that by using angiography (κ = 0.83 vs. 0.39).

## 3. Discussion

In diabetic patients, the BTK arteries are mainly involved with a characteristic symmetrical and multi-segmental stenosis. For this reason, technical success rate of PTA is lower in PAD with diabetes than that in PAD without diabetes [23]. In addition, a study has reported that males were at an increased risk for re-peripheral vascular interventions compared to females [24]. In this study, the clinical success rate of the diabetes and non-diabetes group was 66.7% and 75%, respectively, reflecting that the clinical outcome of the diabetes group was lower, as previously reported. The clinical success rate for males and females was 61.8% and 82.4%, respectively, thus, reflecting that the clinical outcome of males was lower, as previously reported. However, the results of the foot perfusion mapping were not significantly different between the groups, perhaps; the number of patients was too small to perform such a detailed subgroup analysis. In future, if the number of patients is sufficiently large for analysis by various factors, it is expected that more diverse results could be obtained.

The measurement of tissue perfusion is possible using the conventional diagnostic tools; however, recent advances, with the development of surgical and endovascular treatment in PAD, have increased the need for accurate measurement of tissue perfusion and to acquire an image more easily. CT angiography is often used for evaluation of peripheral arterial systems owing to its high spatial resolution and easy standardization. For this reason, there have been many perfusion studies using CT angiography in PAD patients [16,25]. However, the drawback of this method is that since the kidney function is compromised in large numbers of PAD patients, there is an increased renal burden due to the use of iodine contrast agents. Moreover, increased medical costs for examination, and the inability to proceed simultaneously with endovascular or surgical treatment are the other major limitations. To address these shortcomings, a new technique for tissue perfusion based on conventional angiography was developed, and a few reports about the efficacy of 2D perfusion angiography during the PTA procedure were published to reflect the microcirculation of foot [26,27,28]. However, the perfusion image based on 2D angiography, simply provided the color-coded display, in other words, a color representation of vascularity, and with it, three-dimensional (3D) reconstruction was not possible. In addition, the perfusion parameters in 2D angiography were the functional parameters such as AUC, the maximal density peak, time to peak, and so on [26,27,28]. Therefore, cross-sectional image analysis was impossible; and it remains unclear, whether these indirect parameters really reflected the state of the tissue perfusion.

Here, we evaluated the perfusion mapping and parenchymal blood volume, acquired through CBCT-based 3D angiography with cross sectional image analysis during the PTA procedure, as a surrogate marker of clinical outcome. In this study, diagnostic performance of CBCT-based foot perfusion mapping was superior to conventional angiography, with a high consistency of visual assessment among reviewers. In addition, PBV_max_ measured before and after angioplasty, had the ability to predict clinical outcomes. On quantitative analysis, PBV_max_ was significantly increased after angioplasty. Based on these results, CBCT-based foot perfusion mapping was considered useful for assessing treatment response and clinical outcomes. As the technique reconstructs images according to the target element through 3D reconstruction, and presents them in color mapping, the practitioner can intuitively gauge the recanalization results. Moreover, the perfusion values analyzed in this study are the direct parameter of the parenchymal blood volume, therefore, we believe that they are a more accurate reflection of recanalization.

Interestingly, in this study, PBV_max_ of perfusion mapping before angioplasty was also statistically significant for evaluating clinical outcome. Therefore, the lesions with good perfusion in the baseline may have better clinical outcomes after angioplasty.

Accuracy, sensitivity, PPV, and NPV of visual scoring of cone-beam CT-based foot perfusion mapping, was higher than that of angiography for both reviewer 1 and reviewer 2, whereas specificity was relatively lower than that of angiography; thereby suggesting that we wrongly predicted improvement on visual scoring of perfusion mapping for some cases. We think that more accurate results can be achieved, if we can measure oxygenation at the tissue level such as transcutaneous oxygen tension (TcPO2); further studies correlating perfusion mapping and TcPO2 are needed to validate it.

To predict clinical outcome after PTA is important in terms of determining subsequent treatment strategy, especially in CLI patients as the ultimate goal of PTA is to reduce the amputation rate or lower the level of amputation. However, in some cases, the early implementation of proper lower limb amputation may lead to better prognosis of the patient. Therefore, an imaging modality that can provide early insights into the impact of recanalization results would give an opportunity to optimize individual patient management and avoid unnecessary amputation, or treatment delay. Furthermore, the imaging modality needs to be fast, safe, reproducible, and above all, readily accessible at the intervention. In this regard, CBCT-based foot perfusion mapping obtained during the angioplasty provides excellent results during the endovascular treatment, and no additional measuring method is required.

There are several limitations to this study. First, data were obtained retrospectively from a single-center registry with relatively small numbers, which may not address the general application until CBCT-based foot perfusion mapping becomes more widespread. Second, additional use of iodine contrast media for CBCT-based foot perfusion mapping still has limitations for patients with compromised kidney function.

## 4. Conclusions

In conclusion, CBCT-based foot perfusion mapping shows reliable images to evaluate technical success after angioplasty and to predict clinical outcome by both qualitative and quantitative analysis.

## Figures and Tables

**Table 1:** Basic characteristics of 43 patients.

Clinical Characteristics		*N*
Sex	Male	28
	Female	15
Age (year)		68.6 (28–91) *
Underlying disease	Diabetes mellitus	32
	Hypertension	29
	Hyperlipidemia	3
	Smoking	4
	Coronary arterial disease	9
	Hemodialysis	9
Indication	Claudication	17
	Resting pain	1
	Wound	26
	Others	7

* Mean and range are given.

**Table 2:** Correlations between clinical outcome evaluation with visual scoring by imaging modalities after percutaneous transluminal angioplasty and clinical outcome.

Modality	Reviewer	Clinical Outcome	Visual Scoring			
			0	1	2	3
Angiography	1	Improved	4	25	5	1
		Not improved	1	10	3	2
	2	Improved	5	14	15	1
		Not improved	2	6	8	0
Perfusion mapping	1	Improved	2	3	16	14
		Not improved	0	7	2	7
	2	Improved	1	3	16	15
		Not improved	1	6	5	4

**Table 3:** Percutaneous transluminal angioplasty procedures according to the treatment segments.

	POBA	DCB	Bare Metal Stent	DES	Atherectomy
Iliac artery	2				
CFA	1				1
SFA		27	8	2	
Popliteal artery	6	23			
BTK arteries	124				

PTA, percutaneous transluminal angioplasty; POBA, plain old balloon angioplasty; DCB, drug-coated balloon; DES, drug-eluting stent; CFA, common femoral artery; SFA, superficial femoral artery; BTK, below the knee.

**Table 4:** Diagnostic performance of clinical outcome evaluation after percutaneous transluminal angioplasty.

Modality	Reviewer	Sensitivity (95% CI)	Specificity (95% CI)	PPV (95% CI)	NPV (95% CI)	Accuracy (95% CI)
Angiography	1	17.1 (4.7–29.6)	68.8 (46.0–91.5)	54.6 (25.1–84.)	27.5 (13.7–41.3)	33.3 (20.4–46.3)
	2	45.7 (29.2–62.2)	50 (25.5–74.5)	66.7 (47.8–85.5)	29.6 (12.4–46.9)	47.1 (33.4–60.8)
Perfusion mapping	1	85.7 (74.1–97.3)	43.8 (19.4–68.1)	76.9 (63.7–90.2)	58.3 (30.4–86.2)	72.6 (60.3–84.8)
	2	88.6 (78.0–99.1)	43.8 (19.4–68.1)	77.5 (64.6–90.4)	63.6 (35.2–92.1)	74.5 (62.6–86.5)

CI, confidence interval; PPV, positive predictive values; NPV, negative predictive values.

**Table 5:** Prediction of clinical outcome by imaging type and perfusion parameter using multiple logistic regression with C-statistics and odds ratio after adjustment for amputation.

Model	OR (95% CI)	*p* Value	C-Statistic
Angiography	0.56 (0.15–2.12)	0.390	0.75
Cone-beam CT Perfusion mapping	6.54 (1.24–34.38)	0.027	0.81
PBV_max_, before PTA	1.01 (1–1.01)	0.045	0.82
PBV_mean_, before PTA	0.99 (0.96–1.02)	0.625	0.65
PBV_max_, after PTA	1.01 (1.00–1.01)	0.030	0.84
PBV_mean_, after PTA	1.00 (0.98–1.02)	0.701	0.61

OR, odds ratio; CI, confidence interval; CT, computed tomography; PBV_max_, maximum value of parenchymal blood volume; PTA, percutaneous transluminal angioplasty; PBV_mean_, mean value of parenchymal blood volume.

## Data Availability

Data sharing is not applicable to this article.
